# Mapping and Evaluation of Quantitative Trait Loci for Adult-Plant Stripe Rust Resistance in the Sichuan Wheat Cultivar Chuanmai 62

**DOI:** 10.3390/ijms27209196

**Published:** 2026-10-16

**Authors:** Fangjie Yao, Ennian Yang, Jun Li, Wuyun Yang, Manyu Yang

**Affiliations:** 1 Crop Research Institute, Sichuan Academy of Agricultural Sciences, Chengdu 610066, China; yaofangjie@scsaas.cn (F.Y.); lijun@scsaas.cn (J.L.); yangwuyun@126.com (W.Y.); 2 Key Laboratory of Wheat Biology and Genetic Improvement on Southwestern China, Ministry of Agriculture and Rural Affairs of P.R.C., Crop Research Institute, Sichuan Academy of Agricultural Sciences, Chengdu 610066, China; 3 Crop Germplasm Innovation and Genetic Improvement Key Laboratory of Sichuan Province, Crop Research Institute, Sichuan Academy of Agricultural Sciences, Chengdu 610066, China; 4 Institute of Agricultural Resource and Environment, Sichuan Academy of Agricultural Sciences, Chengdu 610066, China; yangennian@126.com

**Keywords:** stripe rust, durable resistance, KASP marker, quantitative trait locus, Chuanmai 62

## Abstract

Stripe rust, caused by *Puccinia striiformis* f. sp. *tritici* (*Pst*), poses a persistent threat to wheat production in China. The Sichuan cultivar Chuanmai 62 (CM62) has exhibited stable adult-plant resistance (APR) for over a decade. To elucidate its genetic basis, we evaluated a recombinant inbred line population (CM62 × Avocet S) across 13 environments and genotyped it using the 55K SNP array. QTL mapping identified six QTL distributed on chromosomes 1BL, 2AS, 2BL, 5AL (two loci), and 5BL. Among these, three major QTL (*QYr.CM62-1BL*, *QYr.CM62-2AS*, and *QYr.CM62-2BL*) were detected. Notably, *QYr.CM62-2BL* was the preeminent locus, explaining 8.97–50.59% of phenotypic variance and exhibiting high stability across 11 of the 13 environments; this locus is closely linked to the previously reported *QYR1* region, although whether it is allelic to *QYR1* remains unclear. *QYr.CM62-2AS* corresponded to *Yr17*/*YrM1225*, while *QYr.CM62-1BL* aligned with *Yr29*. A significant finding was the identification of *QYr.CM62-5BL*, a novel minor-effect QTL mapped to the rearranged 5BL·7BL segment resulting from a 5B/7B reciprocal translocation present in both parents. Furthermore, two Kompetitive Allele-Specific PCR (KASP) markers (*KASP-AX-109507945* and *KASP-AX-109844182*) were developed for *QYr.CM62-2BL*. Polymorphism screening of 328 cultivars confirmed their utility for marker-assisted selection. Collectively, our results demonstrate that the durable APR in CM62 is governed by multiple additive QTL. The newly developed KASP markers, together with the novel *QYr.CM62-5BL* locus and prioritized candidate genes from in silico annotation of the *QYr.CM62-2BL* interval, provide valuable genetic resources for pyramiding resistance alleles and future map-based cloning in wheat breeding programs.

## 1. Introduction

Stripe rust, caused by *Puccinia striiformis* f. sp. *tritici* (*Pst*), is a globally destructive disease of wheat (*Triticum aestivum* L.), leading to substantial yield losses and threatening global food security [1,2]. In China, the southwestern wheat-growing regions, particularly the Sichuan Province, are characterized by climatic conditions conducive to the overwintering and epidemic spread of *Pst*, rendering stripe rust a persistent threat [3]. Northwest Sichuan serves as a pivotal oversummering and overwintering site for *Pst*, representing a hotspot for the emergence of novel virulent races and a primary source of inoculum for spring epidemics in eastern main production zones [4]. Although chemical control remains a common practice, its economic costs and environmental detriments underscore the necessity of genetic resistance as the most sustainable management strategy [4,5]. The rapid evolutionary dynamics of *Pst*, however, frequently lead to the breakdown of race-specific, all-stage resistance (ASR). This highlights the imperative to deploy non-race-specific, durable resistance, notably adult-plant resistance (APR) [6]. APR is typically quantitative, polygenic, and expressed in adult plants under moderately high temperatures, offering long-term and broad-spectrum protection against stripe rust [7].

To date, 88 *Yr* genes have been formally designated [8,9], of which 60 are regarded as conferring ASR and 28 as conferring APR [10]. Numerous additional quantitative trait loci (QTL) for *Pst* resistance have been mapped in diverse genetic backgrounds. ASR genes, such as *Yr5*, *Yr10*, *Yr15*, *Yr17*, *Yr26*, and *Yr27*, typically confer high-level resistance at all growth stages but are often overcome by newly emerged virulent races [11,12]. Notably, the 2025 breakdown of *Yr15* in the UK and continental Europe [13,14] exemplifies this vulnerability and threatens cultivars carrying this widely deployed gene [15]. In contrast, APR genes, such as *Yr18/Lr34/Sr57/Pm38*, *Yr29/Lr46/Sr58/Pm39/Ltn2*, *Yr46/Lr67/Sr55/Pm46*, and *Yr52*, generally provide partial but more durable resistance that is expressed only at later growth stages [16,17,18]. The durability of APR makes it particularly valuable for breeding programs in regions with high stripe rust pressure, such as southwestern China.

In addition to permanently named *Yr* genes, numerous QTL for APR have been identified in recent years [3]. Notable examples include *QYr.cau-2AL* in Chinese landraces [19], *QYr.nmbu.6A* in European spring wheats [20], *QYr.sicau-2BL* and *QYr.sicau-2BS* in Sichuan wheat germplasm [21,22], a major APR QTL on 2AS (temporarily designated *YrR61*) and *QYr.uga-6AS* in US soft red winter wheat [23], and *QYrAs.pau-2A.1* in *Triticum durum*–*Aegilops speltoides* backcross introgression lines [24]. Several of these QTL have been deployed in marker-assisted breeding, though validation across diverse genetic backgrounds remains limited. In the Sichuan wheat-growing region specifically, where stripe rust epidemics are frequent and severe, the identification of stable APR QTL from locally adapted cultivars is of particular practical importance.

Despite these advances, the genetic basis of APR in many Chinese wheat cultivars remains poorly characterized. The resistant cultivar Chuanmai 62 (CM62) exhibits consistent APR across multiple environments for over a decade, yet the underlying QTL have not been systematically mapped. In this study, we constructed a recombinant inbred line (RIL) population derived from CM62 and a susceptible parent to identify and characterize QTL conferring APR to stripe rust. In this study, we aimed to dissect the genetic basis of durable APR in CM62 through integrated genetic mapping and marker development. Specifically, our objectives were to (1) identify and characterize QTL conferring APR in CM62 using an RIL population evaluated across 11 field environments in China, with emphasis on the stability and genetic effects of each locus; (2) compare the mapped QTL intervals with previously reported *Yr* genes and QTL to assess novelty and potential allelism; (3) develop and evaluate breeder-friendly KASP markers for the major QTL *QYr.CM62-2BL*, testing polymorphism in an extended panel of wheat cultivars and advanced lines; and (4) characterize the novel minor-effect QTL *QYr.CM62-5BL* mapped to the rearranged 5BL·7BL segment resulting from a 5B/7B reciprocal translocation, and explore its implications for resistance breeding. Our findings provide new insights into the genetic architecture of durable stripe rust resistance in Chinese wheat germplasm and deliver practical molecular tools for pyramiding resistance alleles in breeding programs.

## 2. Results

### 2.1. Evaluation of Stripe Rust Resistance in Parents and the CM62/Avs Recombinant Inbred Line Population

At the seedling stage in Xindu, both parental lines (CM62 and AvS) exhibited high susceptibility to the composite *Pst* race mixture, displaying disease severity (DS) of 100%. Conversely, under field conditions across 13 environments at the adult-plant stage, CM62 demonstrated stable, high-level resistance (DS, 0–5%), indicative of stable APR. In contrast, AvS remained highly susceptible (DS, 80–100%) across all test sites (Table 1; Table S1).

**Figure 1:**
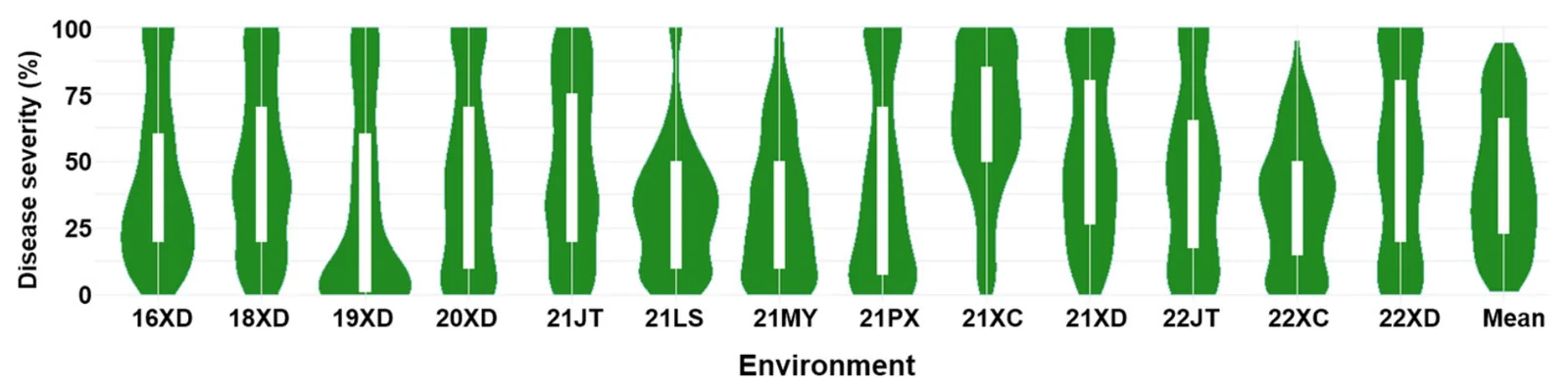
Phenotypic distribution of 281 RILs derived from the CM62 × AvS population across multi-environment trials. Environments 16XD, 18XD, 19XD, 20XD, 21JT, 21LS, 21MY, 21PX, 21XC, 21XD, 22JT, 22XC, and 22XD correspond to 13 experimental sites spanning Xindu, Jitian, Linshui, Mianyang, Pixian, and Xichong districts of Sichuan Province from 2016 to 2022. “Mean” represents the mean disease severity across all 13 environments.

**Figure 2:**
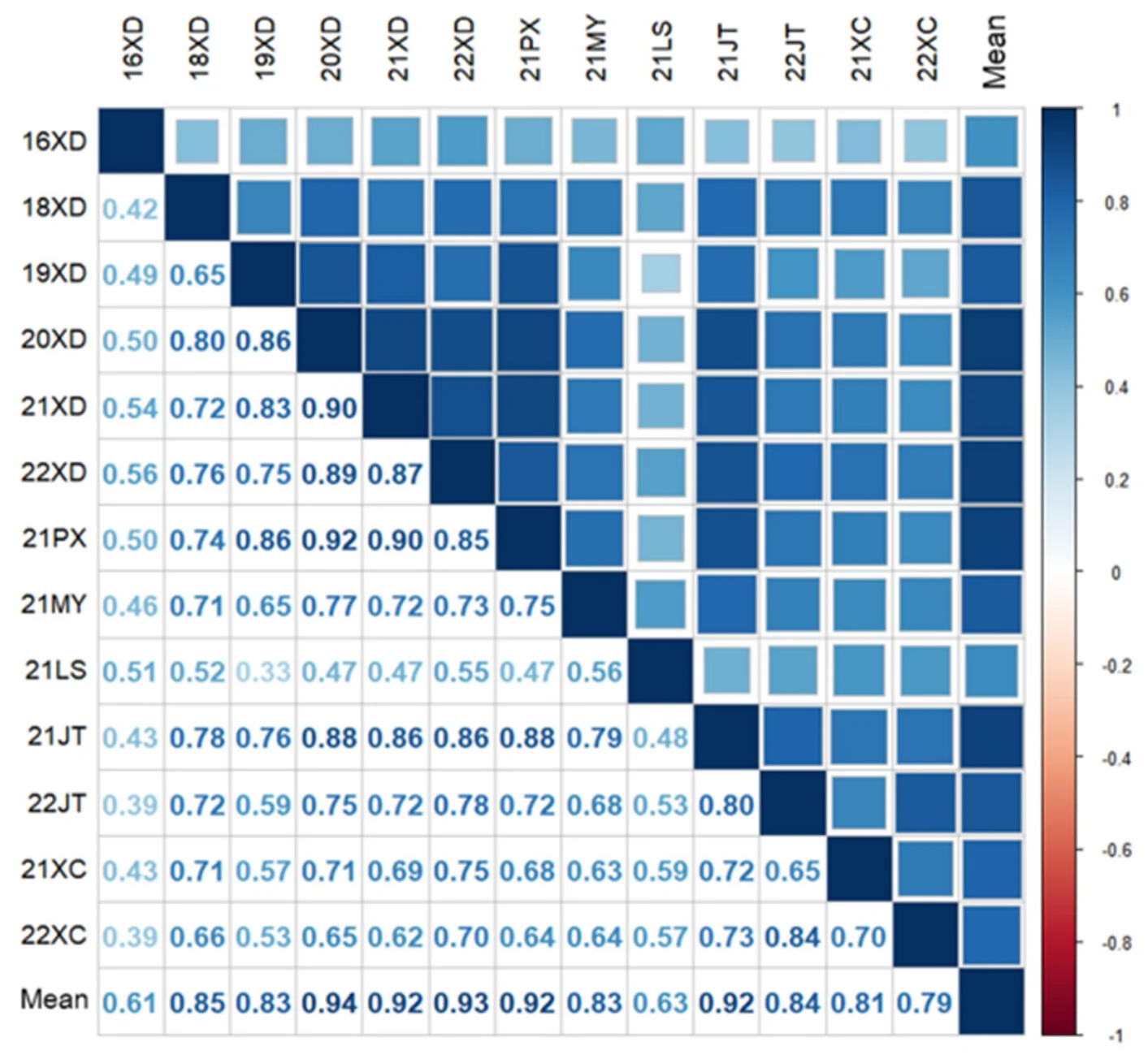
Pairwise correlations of disease severity across environments in the CM62/AvS RIL population. Correlation coefficients are visualized using a gradient from positive (blue) to negative (red), with color saturation and square size proportional to the absolute correlation coefficients. The series 16XD, 18XD, 19XD, 20XD, 21XD, 22XD, 21PX, 21MY, 21LS, 21JT, 22JT, 21XC, and 22XC denote disease severity datasets evaluated from 13 field trial sites across the Xindu, Pixian, Mianyang, Linshui, Jitian, and Xichong districts of Sichuan Province, covering the period from 2016 to 2022. “Mean” indicates the average disease severity aggregated across all 13 tested environments. Notably, the Pearson correlation coefficients for the adult-plant stage exhibit highly significant *p*-values (*p* < 0.001).

Within the 281 RILs, the DS values ranged from 0 to 100% across environments (Table 1; Figure 1). The continuous phenotypic distribution supports the hypothesis that stripe rust resistance in CM62 is quantitatively inherited and likely governed by multiple genes or QTL. Broad-sense heritability (*H^2^*) for DS was estimated at 0.68, indicating that genetic factors predominantly accounted for the phenotypic variation in resistance, with moderate environmental influence (Table 1). Significant positive correlations (*p* < 0.001) were observed among DS datasets across all 13 environments and the mean DS, with correlation coefficients ranging from 0.33 to 0.94 (Figure 2). Field trials at Xindu, Pixian, and Jitian were artificially inoculated with a mixture of *Pst* urediniospores, whereas those at Linshui, Mianyang, and Xichong were maintained under natural epidemic conditions. Consequently, DS varied considerably across the 13 environments (CV ranging from 0.42 to 1.07), reflecting differences in epidemic pressure among locations and years, as evidenced in both Figure 1 and Figure 2. Detailed DS records for both parents and the 281 RILs across all tested environments are presented in Table S1.

### 2.2. Genetic Linkage Map Construction and the ND-FISH Analysis

A total of 20,179 polymorphic SNP markers were initially genotyped using the Affymetrix Axiom Wheat55K SNP array. After applying quality filters (MAF ≥ 30% and missing rate ≤ 20%), 14,210 markers were retained for further analysis. These markers were consolidated into 2562 non-redundant recombination bins, which served as the framework for genetic map construction. The resulting linkage map comprised 2562 loci distributed across 21 linkage groups, corresponding to the full complement of wheat chromosomes. The total genetic length was 4101.44 cM, yielding an average interval of 1.60 cM between adjacent markers (Table S2). The genotype matrix for the CM62/AvS RIL population is presented in Table S3.

**Figure 3:**
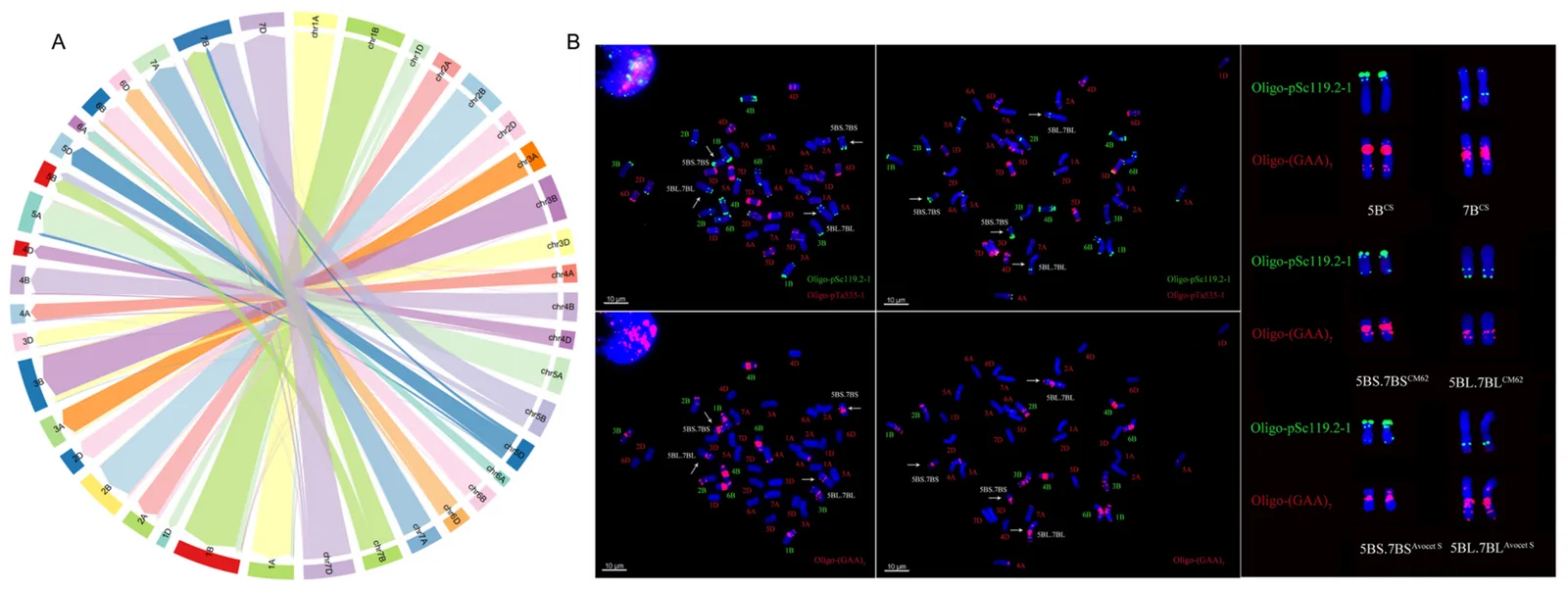
Cytogenetic characterization and molecular mapping of the 5B/7B reciprocal translocation in the CM62/AvS RIL population. (**A**) Circos plot showing the concordance between physical positions (chr1A-chr7D in the right part, based on IWGSC RefSeq v1.0) and genetic linkage maps (1A-7D in the left part). The purple and green ribbons bridging chromosomes 5B and 7B highlight the reciprocal exchange of chromosomal segments. (**B**) ND-FISH validation of the homozygous 5B/7B translocation in parental lines using Oligo-pSc119.2-1 (green) and Oligo-pTa535-1/Oligo-(GAA)_7_ (red) probes. White arrows point to the characteristic 5BS·7BS and 5BL·7BL translocation chromosomes. Scale bar, 10 μm.

Analysis of the genetic linkage map identified a clear translocation between chromosomes 5B and 7B relative to their physical positions, which was visualized using a Circos plot (Figure 3A). ND-FISH analysis confirmed the presence of 5BS·7BS and 5BL·7BL translocation chromosomes in both CM62 and AvS (Figure 3B). The consistent manifestation of this translocation in the RIL linkage map indicates that the reciprocal translocation is transmitted stably to the RIL population and maintained as a fixed chromosomal structure.

### 2.3. QTL Analysis

**Figure 4:**
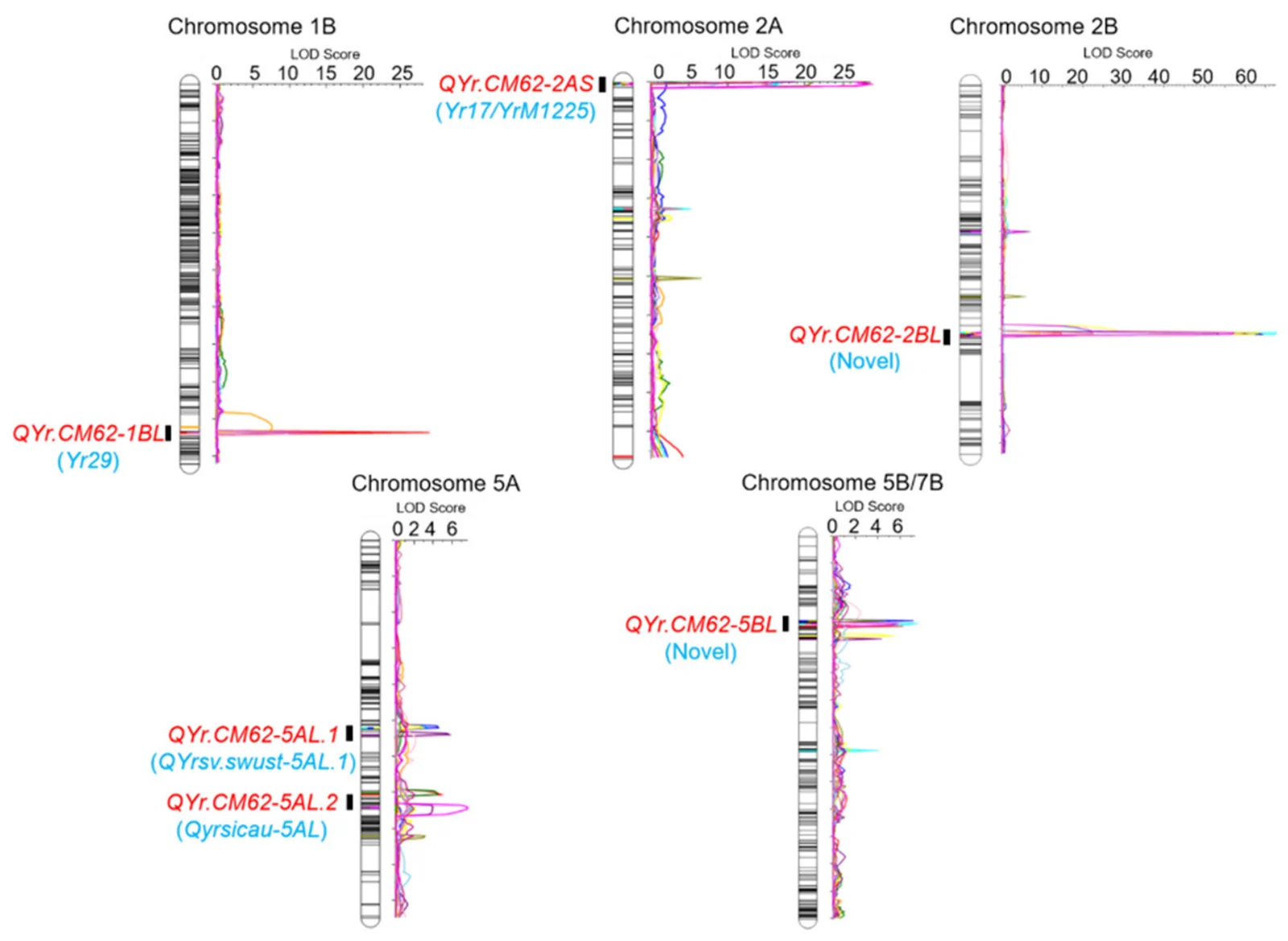
Graphical displays of locations of QTL for stripe rust resistance across 13 environments and mean data for the CM62/AvS RIL population. QTL labels in parentheses (blue) denote previously reported loci putatively corresponding to the same genomic regions identified in this study.

Inclusive composite interval mapping (ICIM) identified six stable QTL for stripe rust resistance, consistently detected across ≥ 4 of the 13 environments and their mean (Figure 4). Designated *QYr.CM62-1BL*, *QYr.CM62-2AS*, *QYr.CM62-2BL*, *QYr.CM62-5AL.1*, *QYr.CM62-5AL.2*, and *QYr.CM62-5BL*, these loci were mapped to chromosomes 1BL, 2AS, 2BL, 5AL (two loci), and 5BL, respectively (Table 2). All were donated by the resistant parent CM62, exhibiting negative additive effects, with individual PVEs ranging from 1.34% to 50.59%.

Notably, *QYr.CM62-2BL* emerged as the preeminent QTL, which exhibited the highest LOD score, explaining up to 50.59% of the PVE, and was detected in 11 of the 13 environments. *QYr.CM62-1BL* (mean PVE 12.34%) and *QYr.CM62-2AS* (mean PVE 12.07%) were also classified as major QTL. The remaining loci (*QYr.CM62-5AL.1*, *QYr.CM62-5AL.2*, and *QYr.CM62-5BL*) were minor, with mean PVEs of 1.77–3.40%. Furthermore, *QYr.CM62-2BL* may represent either a novel allele at the *QYR1* locus or a tightly linked QTL, whereas *QYr.CM62-5BL* represents a novel locus within the 5B/7B reciprocal translocation region. The other four QTL co-localized with known resistance loci: *QYr.CM62-1BL* with *Yr29*, *QYr.CM62-2AS* with *Yr17/YrM1225*, *QYr.CM62-5AL.1* with *QYrsv.swust-5AL.1*, and *QYr.CM62-5AL.2* with *Qyrsicau-5AL*, respectively.

### 2.4. Effects of Six QTL and Their Combinatorial Interactions

**Figure 5:**
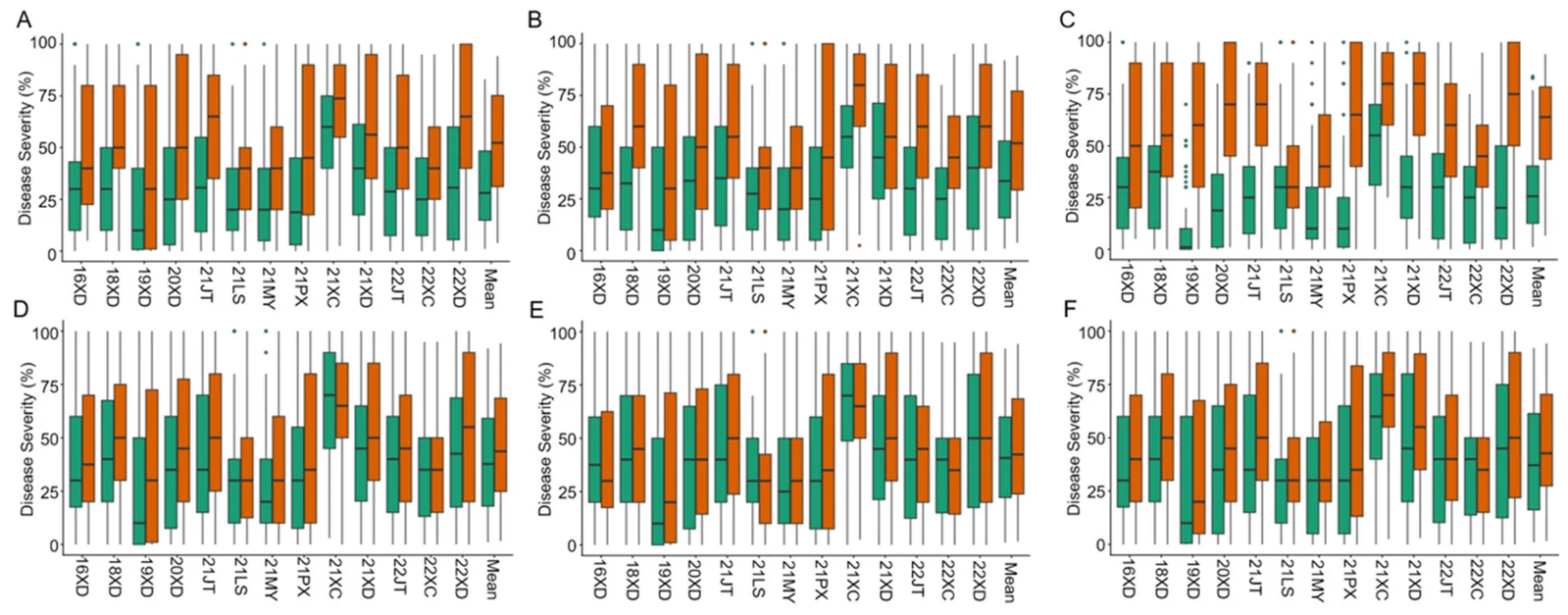
Comparison of disease severity between CM62/AvS RILs carrying (green bars) versus those lacking (orange bars) the target allele across 13 environments and the overall mean. (**A**–**F**) represent the six identified QTL: *QYr.CM62-1BL*, *QYr.CM62-2AS*, *QYr.CM62-2BL*, *QYr.CM62-5AL.1*, *QYr.CM62-5AL.2*, and *QYr.CM62-5BL*. Error bars indicate SE. Individual dots above the boxes represent outliers. Student’s *t*-tests revealed that the target allele significantly mitigated disease severity across all environments. Except for the 21LS environment (*p* < 0.05), the reduction in disease severity was highly consistent and statistically robust (*p* < 0.001) across the remaining twelve environments. Environments 16XD, 18XD, 19XD, 20XD, 21JT, 21LS, 21MY, 21PX, 21XC, 21XD, 22JT, 22XC, and 22XD correspond to 13 experimental sites spanning Xindu, Jitian, Linshui, Mianyang, Pixian, and Xichong districts of Sichuan Province from 2016 to 2022. “Mean” represents the mean disease severity across all 13 environments.

To evaluate the phenotypic effects of the six QTL identified in this study, we assessed the disease severity of 281 RILs across 13 environments and the overall mean in the CM62/AvS RIL population. As shown in Figure 5, all six QTL consistently and significantly reduced disease severity compared with the non-target controls (*p* < 0.001 except for 21LS (*p* < 0.05), Student’s *t*-test). Across the 13 environments, the resistance QTL exhibited significant and context-dependent effects on disease severity. For each QTL, genotypes carrying the resistance allele (green bar) consistently showed markedly lower disease severity compared to those without the allele (orange bar), confirming the effectiveness of these QTL in conferring resistance (Figure 5A–F). For instance, in highly conducive environments such as 21XC, 21XD, and 22XC, the susceptibility (high orange bars) was extremely severe, whereas the presence of the resistance QTL (green bars) substantially suppressed the disease progression. Based on the phenotypic data across the 13 environments, *QYr.CM62-2BL* (Figure 5C) demonstrates the most superior and stable resistance effect, making it the top priority candidate for subsequent marker-assisted breeding programs.

Moreover, the analysis of QTL combinations revealed a clear trend: genotypes carrying multiple resistance QTL demonstrated significantly lower disease severity and greater stability across environments compared to fewer QTL carriers. However, the magnitude of resistance varied considerably among different environments, highlighting strong QTL-by-environment interactions. Notably, the mean values across environments further confirmed that the cumulative effect of these resistance alleles provided robust and durable protection against stripe rust (Figure S1).

### 2.5. Development of KASP Markers for Molecular-Assisted Selection

**Figure 6:**
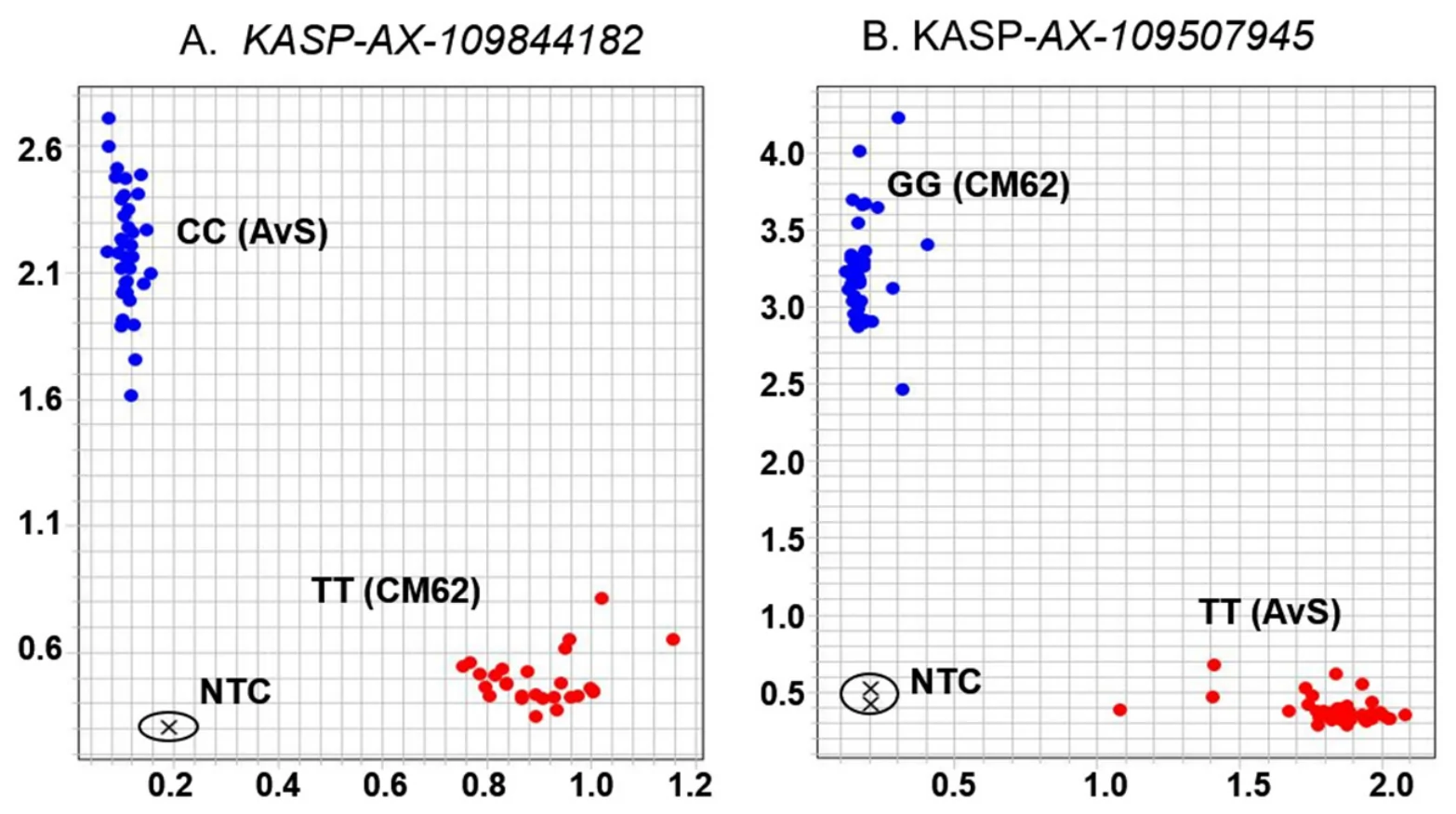
Scatter plots of selected KASP assays illustrating genotype clustering of part CM62/AvS RILs. A and B depict KASP assay profiles for *KASP-AX-109844182* and *KASP-AX-109507945*, respectively, which are genetically linked to *QYr.CM62-2BL* in the RIL population.

Given that *QYr.CM62-2BL* was postulated as the most major-effect and stable locus in this population, closely linked flanking SNP markers were selected for conversion into KASP assays. Two flanking markers (*KASP-AX-109844182* and *KASP-AX-109507945*) were successfully genotyped and exhibited clear polymorphism between the parental lines CM62 and AvS (Figure 6; Table S4). Evaluation across the RIL population confirmed that these KASP markers yielded identical genotypic calls to their corresponding SNP loci in individual RILs (Table S5). To assess marker specificity and polymorphism for MAS, the two flanking KASP markers were further evaluated in a diverse wheat panel comprising 157 cultivars (and advanced lines) developed in Sichuan and 171 CIMMYT germplasm accessions (Table S6). Resistance-conferring alleles at both loci were identified in 21.02% of Sichuan-bred accessions, compared to only 4.09% in the CIMMYT germplasm. This disparity indicates that there remains considerable scope for broader deployment of *QYr.CM62-2BL* in current breeding programs despite its strong resistance to stripe rust. The tightly linked KASP markers developed herein offer promising tools for MAS in regional wheat breeding programs in Sichuan Province, pending large-scale validation of marker–phenotype associations in independent germplasm.

### 2.6. The 5B/7B Reciprocal Translocation in the CM62/Avs RIL Population

Building on the cytogenetic and linkage evidence presented in Section 2.2, we further defined the precise breakpoints. Cytogenetic analyses confirmed that CM62 carries the 5BS·7BS and 5BL·7BL translocation chromosomes, consistent with previous findings [25,26], and further demonstrated that AvS also harbors the 5B/7B reciprocal translocation (Figure 3B). In this study, a high-density genetic map was constructed for the CM62/AvS RIL population using the Wheat 55K SNP array. Comparative analysis between genetic and physical maps revealed that the breakpoint at 59.56 Mb on 5BS was juxtaposed with the breakpoint at 162.62 Mb on 7BS, constituting a 5BS·7BS translocation chromosome. Concurrently, the breakpoint at 377.12 Mb on 5BL fused with the breakpoint at 387.44 Mb on 7BL, forming a 5BL·7BL translocation chromosome (Figure 3; Table S7). These results provide molecular evidence for a whole-arm reciprocal translocation between chromosomes 5B and 7B. Given that both parents are homozygous for this translocation, the RIL population stably inherits the translocation configuration, and the high-density genetic map constructed herein serves as a molecular-level corroboration of this translocation event.

## 3. Discussion

CM62 was bred through shuttle breeding in collaboration with CIMMYT, derived from a cross between the French cultivar Fr3 and the elite line SW1862. This cultivar has demonstrated durable, high-level resistance to stripe rust under field conditions for over a decade. In this study, six stripe rust resistance QTL on chromosomes 1BL, 2AS, 2BL, 5AL (two QTL), and 5BL were mapped using the CM62/AvS RIL population. Of these, *QYr.CM62-1BL*, *QYr.CM62-2AS*, and *QYr.CM62-2BL* were designated as major-effect QTL, while *QYr.CM62-5AL.1*, *QYr.CM62-5AL.2*, and *QYr.CM62-5BL* were categorized as minor-effect QTL (Table 2; Figure 4). Comparative analysis with previously reported *Yr* genes/QTL revealed that five loci co-localized with known resistance genes. Additionally, *QYr.CM62-5BL*, located within the 5B/7B reciprocal translocation, constitutes a putative novel resistance locus. Notably, *QYr.CM62-2BL* accounted for 8.97–50.59% of the PVE across 11 of 13 tested environments and was the most prominent major-effect and stable locus within this population. Fine-mapping of *QYr.CM62-2BL* using a larger segregating population is currently underway in our laboratory. Once the interval is narrowed, qPCR-based expression analysis of prioritized candidate genes will be performed to validate their involvement in stripe rust resistance.

### 3.1. Identification of QYr.CM62-1BL as Yr29

*QYr.CM62-1BL* was localized to a 660.43–670.39 Mb interval on chromosome 1BL, flanked by molecular markers *AX 111240459* and *AX 111450994*, and explained 6.67–19.34% of the PVE (Table 2; Figure 4). This QTL coincided precisely with a well-characterized resistance hotspot harboring the pleiotropic gene *Yr29/Lr46/Sr58/Pm39* [3]. *Yr29/Lr46/Sr58/Pm39* confers durable, broad-spectrum resistance against multiple foliar pathogens, including various rust species and powdery mildew [3,27,28,29]. Notably, *Yr29/Lr46/Sr58/Pm39* has provided partial APR to leaf and stripe rust for over six decades and remains integral to CIMMYT’s long-term disease resistance breeding strategy. Given that the male parent of CM62, SW1862, was derived from CIMMYT germplasm, the *QYr.CM62-1BL* locus identified herein is highly likely to represent an introgression from CIMMYT genetic resources.

### 3.2. Identification of QYr.CM62-2AS as Yr17/YrM1225

*QYr.CM62-2AS* was consistently mapped to a 0.29–24.88 Mb interval within the distal region of the short arm of chromosome 2AS across 11 of 13 tested environments and in the overall mean data. Delimited by the flanking markers *AX-110085687* and *AX-109973606*, this QTL explained 2.98–20.01% of the PVE (Table 2, Figure 4). This APR locus co-localized with the ASR gene *Yr17* [30], corroborating previous findings by Liu et al. [31] and Mu et al. [32]. The mixed *Pst* races used in this study included CYR34, which is virulent against *Yr17*; accordingly, the Avocet near-isogenic line (NIL) carrying *Yr17* exhibited high susceptibility (DS = 100%) under our inoculation conditions. Thus, the seedling susceptibility observed in CM62 (also DS = 100%) cannot be used to exclude the presence of *Yr17*, as the inoculation results simply reflect the virulence of the prevailing race rather than the absence of the gene. Given that *Yr17* is tightly linked to the high-temperature adult-plant resistance gene *YrM1225* on chromosome 2AS [33], and our QTL interval (0.29–24.88 Mb) could not resolve the two loci, whether *QYr.CM62-2AS* corresponds to *Yr17*, *YrM1225*, or a distinct allele remains to be determined.

### 3.3. Identification of QYr.CM62-2BL as a Novel Allele or a Tightly Linked QTL at the QYR1 Locus

*QYr.CM62-2BL* was stably mapped to a 682.72–696.35 Mb interval within the distal region of chromosome 2BL, explaining 8.97–50.59% of the PVE across 11 of 13 evaluated environments and overall mean data (Table 2, Figure 4). Chromosome 2B represents one of the most gene-dense regions for wheat stripe rust resistance in the wheat genome, harboring over 50 cataloged resistance genes and QTL clustered within at least five distinct regions [3]. Notably, *Yr5*, *Yr7*, and *YrSP* co-localize to an identical physical interval (685.265–685.270 Mb) and encode nucleotide-binding leucine-rich repeat proteins [34]. While *Yr5* and *Yr7* are established alleles, *YrSP* constitutes a truncated allele of *Yr5* [34]. Given that *Yr5*, *Yr7*, and *YrSP* function as ASR genes, whereas *QYr.CM62-2BL* confers APR, they are likely distinct. Consistent with this, both parents and the RIL population were highly susceptible at the seedling stage (DS = 100%), confirming that CM62 does not carry functional *Yr5*/*Yr7*/*YrSP* alleles (Table 1, Figure 1). Within this genomic region, *QYR1*, derived from the cultivar Camp Remy, was previously mapped to a 672.08–685.76 Mb interval flanked by markers *gwm501* and *gwm47*, accounting for 46% of the total phenotypic variance [35]. The substantial overlap between the *QYR1* (672.08–685.76 Mb) and *QYr.CM62-2BL* (682.72–696.35 Mb) intervals, specifically a ~3.0 Mb shared segment (682.72–685.76 Mb), strongly suggests that these loci are either allelic or represent the same genetic entity. However, the distal extension of *QYr.CM62-2BL* beyond the *QYR1* interval indicates that *QYr.CM62-2BL* may either represent a novel allele at the *QYR1* locus or a previously uncharacterized, tightly linked resistance gene on chromosome 2BL. The precise relationship between *QYr.CM62-2BL* and *QYR1* will be elucidated in future studies.

To explore the potential genetic basis of *QYr.CM62-2BL*, we performed in silico annotation of the Chinese Spring reference genome within the mapped interval (chr2B: 682.72–696.35 Mb, IWGSC RefSeq v1.0) (Table S8). This ~13.6 Mb region contains 35 high-confidence genes, including a prominent nucleotide-binding leucine-rich repeat (NLR) cluster proximal to the *Yr5/Yr7* anchor (683.0–686.1 Mb) and several distal defense-related candidates (leucine-rich repeat receptor-like kinases (LRR-RLKs), pathogenesis-related protein 1 (PR1), and transcription factors (TFs)). The enrichment of NLR and defense-related genes (e.g., LRR-RLK, PR1, TFs) within this interval provides a plausible biological basis for the APR phenotype, suggesting *QYr.CM62-2BL* may represent a composite resistance locus. High-priority distal candidates will be validated via qPCR after fine-mapping.

### 3.4. Co-Identification of QYr.CM62-5AL.1 and QYr.CM62-5AL.2 with Known Loci

*QYr.CM62-5AL.1* and *QYr.CM62-5AL.2* were identified as minor QTL localized within the 503.27–528.48 Mb and 565.55–573.62 Mb intervals on the long arm of chromosome 5A, explaining 1.34–2.26% and 1.57–3.19% of the PVE, respectively (Table 2, Figure 4). To date, over 16 QTL and *Yr* genes have been documented on chromosome 5A [3,21,36]. Notably, *QYrsv.swust-5AL.1* [36] was mapped to the 512.23–519.89 Mb region flanked by *IWA5118* and *IWB60872* on 5AL, which overlaps positionally with *QYr.CM62-5AL.1*. Furthermore, *Qyrsicau-5AL* [21], a QTL significantly associated with marker *AX-109907430* near 566.77 Mb and derived from a Sichuan wheat panel, resides within the genomic interval of *QYr.CM62-5AL.2*.

### 3.5. Identification of QYr.CM62-5BL on the Rearranged Chromosomes 5BL·7BL

*QYr.CM62-5BL* was identified as a minor QTL localized within the 622.55–644.85 Mb physical interval on chromosome arm 5BL, accounting for 2.38–4.85% of the PVE across 5 of 13 evaluated environments as well as in the mean dataset (Table 2, Figure 4). As described above, both parents are homozygous for the 5B/7B reciprocal translocation, with breakpoints at 377.12 Mb on 5BL and 387.44 Mb on 7BL. Because recombination proceeds normally in translocation homozygotes, SNPs within the rearranged segment segregated and recombined regularly in the RIL population, permitting QTL mapping. The physical coordinates reported here are based on the Chinese Spring reference genome (RefSeq v1.0); in the translocation background, this 5BL segment (>377.12 Mb) resides on the derived chromosome carrying the 5BL distal segment, and the observed discrepancy between its genetic (61.83–66.47 cM) and physical positions reflects the rearrangement (Figure 3). While several stripe rust resistance QTL have been reported on 5BL, they are genetically distinct from *QYr.CM62-5BL.* Specifically, *QYr.AYH-5BL* (521.71–539.02 Mb) was identified in the landrace ‘Anyuehong’ [37]; *QYr.caas-5BL.2* (386–605.5 Mb), flanked by *Xwmc215* and *Xbarc142*, originated from ‘Libellula’ [38]; *QYr.sun-5B* from durum wheat ‘Wollaroi’ peaks near 605 Mb [39]; and *QYrSW.wgp-5B.3* maps to 620.02 Mb, proximal to *QYr.CM62-5BL* [31].

The 5BS·7BS/5BL·7BL reciprocal translocation, first reported in Cappelle-Desprez [40], has long been associated with durable APR to stripe rust [41]. Mallard et al. [42] mapped *QYr.inra-5BL.2* to the 8.18–13.18 Mb region on the translocated segment of 5BS, which is distinct from the locus reported here. Therefore, *QYr.CM62-5BL* represents a novel resistance locus identified on the 5B/7B translocation background. This translocation has been extensively exploited in global wheat breeding programs due to its superior disease resistance, broad adaptability, and minimal pleiotropic effects on agronomic traits [26,40,41,43]. The identification of *QYr.CM62-5BL* provides a valuable genetic resource for marker-assisted gene pyramiding in elite cultivars already harboring this advantageous translocation.

### 3.6. Pyramiding of Multiple QTL/Genes Enhances Durable Disease Resistance

Pyramiding multiple QTL/genes represents a robust strategy for achieving durable disease resistance in wheat. In this study, we observed that RILs carrying a greater number of resistance loci consistently exhibited enhanced resistance levels (Figure S1). This finding is consistent with previous reports validating the efficacy of gene pyramiding in wheat landraces and cultivars [44,45,46,47,48,49]. Notably, the major locus *QYr.CM62-2BL* identified in CM62 conferred stable, high-level resistance to stripe rust across all tested environments, with highly significant differences between RILs carrying versus lacking this QTL (Figure 5C). To further dissect the contribution of this locus, we identified 11 single seed descent (SSD) lines carrying *QYr.CM62-2BL* as the sole resistance allele (homozygous resistant at *KASP-AX-109844182* and *KASP-AX-109507945*; homozygous susceptible at all five other mapped QTL; Tables S1, S3 and S5; yellow highlighting indicates these lines in each table). Interestingly, these single-locus lines exhibited variable field responses with mean DS ranging from 17.29% to 83.46% (Table S1), despite *QYr.CM62-2BL* explaining 8.97–50.59% of the phenotypic variance. This phenotypic variation highlights the role of modifier genes and genetic background in resistance expression, reinforcing that the durable, high-level field resistance of CM62 is achieved through pyramiding of multiple complementary loci rather than any single QTL acting alone. These 11 single-locus lines provide valuable genetic resources for fine-mapping and map-based cloning of *QYr.CM62-2BL*. Furthermore, based on *QYr.CM62-2BL*, two KASP markers (*KASP-AX-109844182* and *KASP-AX-109507945*) were developed, providing reliable tools for future MAS and accelerating the deployment of durable resistance in wheat breeding programs.

## 4. Materials and Methods

### 4.1. Plant Materials

An RIL population of 281 F_6_ individuals was developed in 2015 from a cross between CM62, selected for its durable APR to predominant Chinese *Pst* races since its release, and the susceptible Australian spring wheat cultivar Avocet S (AvS). The population was advanced to the F_6_ generation using the single-seed descent method. All RILs and parental lines were maintained for phenotypic evaluation. The highly susceptible cultivar Chuanmai 24 served dual roles as a border-row infector and an internal susceptible control. A diverse germplasm panel comprising 157 Sichuan cultivars and elite breeding lines, alongside 171 CIMMYT introductions, was employed to validate the user-friendly markers developed for the identified QTL.

### 4.2. Field Trials and Phenotyping

Stripe rust responses of the 281 RILs and the two parents were evaluated from 2016 to 2022: Xindu (30°49′22.9″ N, 104°10′32.4″ E), Pixian (30°47′52.8″ N, 103°53′35.0″ E), Mianyang (31°27′45.8″ N, 104°44′46.8″ E), Linshui (30°20′14.9″ N, 106°55′34.2″ E), Jitian (30°34′39.1″ N, 103°55′16.5″ E), and Xichong (31°0′2.7″ N, 105°53′9.0″ E). Individual trial environments were designated by a combination of year and site code: 16XD, 18XD, 19XD, 20XD, 21XD, 21JT, 21LS, 21MY, 21PX, 21XC, 22JT, 22XC, and 22XD. These six sites are situated in the western Sichuan Basin and surrounding hilly regions, characterized by a cool and humid subtropical climate favorable for stripe rust development. During the growing season (October–May), mean temperatures ranged from 12.48 to 14.03 °C, mean relative humidity ranged from 73.88 to 86.50%, and cumulative precipitation ranged from 167.10 to 559.10 mm across the 13 environments. Climatic parameters for the 13 environments during the growing season, including mean temperature (°C), mean relative humidity (%), and cumulative rainfall (mm) for each site and year, are presented in Table S9.

Trials were arranged in a randomized complete block design with two replicates per environment. Sowing occurred in late October or early November. Each RIL was planted in a single 1.0 m row with 0.25 m inter-row spacing, sowing 20–30 seeds per row. AvS was planted every 20 rows as an internal susceptible check, while Chuanmai 24 was sown along plot perimeters as an infector row. Trials were managed under rain-fed conditions without supplemental irrigation. At the tillering stage (Zadoks GS 23–25), plots at Xindu, Pixian, and Jitian were artificially inoculated with a 1:20 (*w/w*) mixture of *Pst* urediniospores and talc. The inoculum consisted of a 1:1:1 mixture of prevalent Chinese *Pst* races CYR32, CYR33, and CYR34 (Gansu Academy of Agricultural Sciences) [50]. The avirulence/virulence formulae of the *Pst* races are provided in Table S10. Plots at Linshui, Mianyang, and Xichong relied on natural epidemic conditions. DS was scored twice at approximately one-week intervals once the flag leaves of the susceptible control (AvS) reached full susceptibility (80–100% DS), and the maximum DS value was used for each genotype. DS was visually estimated as the percentage of symptomatic leaf area on a continuous 0–100% scale [51].

### 4.3. Statistical Analysis of Phenotypic Data

Frequency distributions of DS and QTL effect plots were generated using the ggplot2 package in R v4.0.3 [52]. Descriptive statistics, including minimum, maximum, mean, standard deviation, and coefficient of variation, were calculated using Excel 2021 (Microsoft Corp., Redmond, WA, USA). Pearson’s correlation coefficients (*r*) were employed to assess pairwise environmental correlations and visualized using the corrplot package in R v4.0.3 [53]. Broad-sense heritability (*H^2^*) was estimated in IciMapping v4.2 using the formula *H^2^* = σ^2^_G_/[σ^2^_G_ + (σ^2^_E_ + σ^2^_ExG_ + σ^2^_e_)/n], where σ^2^_G_, σ^2^_E_, σ^2^_E×G_, and σ^2^_e_ denote the variance components for genotype, environment, genotype-by-environment interaction, and residuals, respectively, and n is the number of environments. Genotype, environment, and their interaction were treated as random factors in the mixed model. Chromosomal translocation was visualized using the circlize package in R v4.0.3 [54].

### 4.4. DNA Extraction and Molecular Marker Genotyping

Leaf tissues were harvested from individual seedlings of the RIL and polymorphism analysis populations grown in the field. Genomic DNA was isolated using a modified CTAB protocol [55]. DNA integrity and concentration were verified by 1.0% agarose gel electrophoresis and spectrophotometric quantification (NanoDrop ND-1000; Thermo Scientific, Wilmington, DE, USA). The 281 RILs and parental lines were genotyped using the Affymetrix Wheat55K SNP array at CapitalBio Corporation (Beijing, China; http://www.capitalbio.com).

### 4.5. Cytogenetic Analysis

To verify the previously documented reciprocal translocation between chromosomes 5B and 7B in CM62 and to characterize these chromosomes within the AvS genetic background, non-denaturing fluorescence in situ hybridization (ND-FISH) was conducted on metaphase chromosome spreads. The oligonucleotide probes, including Oligo-pSc119.2-1, Oligo-pTa535-1, and Oligo-(GAA)7, were synthesized by Sangon Biotech (Shanghai) Co., Ltd. (Shanghai, China) and used for ND-FISH analysis. The sequences of these probes were adopted from Tang et al. [25] (Table S11). Oligo-pSc119.2-1 was 5′-end-labeled with 6-carboxyfluorescein (6-FAM; Thermo Fisher Scientific, Waltham, MA, USA; green), Oligo-pTa535-1 with 6-carboxytetramethylrhodamine (TAMRA; Thermo Fisher Scientific; red), and Oligo-(GAA)_7_ with Cy5 (GE Healthcare, Chicago, IL, USA; far-red). ND-FISH procedures strictly followed the protocols described by Fu et al. [56] and Yang et al. [57]. Probe concentrations per slide were applied as specified by Tang et al. [25]. Chromosomal signals were captured using a Leica DM4B epifluorescence microscope (Leica Microsystems GmbH, Wetzlar, Germany) and subsequently processed with Adobe Photoshop CC 2021 (Adobe Inc., San Jose, CA, USA).

### 4.6. Genetic Map Construction and QTL Analysis

SNPs with a minor allele frequency (MAF) ≥ 30% and a missing rate ≤ 20% were retained for analysis. Redundant markers were then removed using the BIN function in QTL IciMapping v4.2. Genetic linkage maps were constructed with the MAP function in the same software. For QTL analysis, DS data from each of the 13 environments, as well as the mean data (Mean) across all environments, were utilized. Inclusive composite interval mapping (ICIM) was performed via the BIP function, with a scanning step of 1.0 cM. The threshold for entering variables in the stepwise regression was set at *p* = 0.001. Significant QTL were declared based on logarithm of odds (LOD) thresholds determined through 1000 permutation tests at a type I error rate of 0.05. The confidence interval for each QTL was defined by a one-LOD drop from the peak position. QTL detected in at least four environments were considered stable. The physical positions of flanking SNPs were determined by aligning their sequences to the Chinese Spring reference genome (RefSeq v1.0) using BLAST (https://blast.ncbi.nlm.nih.gov/, accessed on 11 June 2025), enabling comparison with previously reported genes and QTL [58].

### 4.7. Development of KASP Markers for QTL Validation

To validate the identified QTL, tightly linked SNP markers from the array were converted into KASP markers using the PolyMarker bioinformatics pipeline (https://www.polymarker.info/). Allele-specific forward primers were tailed at the 5′ end with the FAM (5′-GAAGGTGACCAAGTTCATGCT-3′) or HEX (5′-GAAGGTCGGAGTCAACGGATT-3′) universal fluorescence sequences. Working primer stocks were diluted to 100 µM. Primer mixes (100 µL final volume) comprised 12 µL of each forward primer, 30 µL of the common reverse primer, and 46 µL of nuclease-free water. KASP assays were conducted in 96-well plates on a Veriti Thermal Cycler (Applied Biosystems; Thermo Fisher Scientific, Waltham, MA, USA) in a 10 µL reaction volume containing 2.0 µL genomic DNA (50 ng/µL), 2.0 µL primer mix, 4.5 µL 2× HiGeno Probe Mix A (JiaCheng Biotech, Beijing, China), and 1.5 µL nuclease-free water. Thermal cycling conditions included an initial denaturation at 95 °C for 10 min; 10 touchdown cycles (95 °C for 20 s, 61–55 °C for 40 s with a 0.6 °C decrement per cycle); and 38 amplification cycles (95 °C for 20 s, 55 °C for 40 s). Endpoint fluorescence was acquired using a QuantStudio™ 6 Flex Real-Time PCR System (Applied Biosystems™; Thermo Fisher Scientific, Waltham, MA, USA). Polymorphisms of the developed KASP markers were initially validated using the parental lines and the 281 RILs. Subsequently, to assess their applicability in MAS, a diverse panel of 157 Sichuan wheat cultivars (advanced lines) and 171 CIMMYT germplasm accessions was genotyped.

## 5. Conclusions

This study deciphers the genetic architecture of durable APR to stripe rust in the elite Sichuan cultivar CM62. We identified six QTL across 13 environments, including three major loci (*QYr.CM62-1BL*, *QYr.CM62-2AS*, and *QYr.CM62-2BL*) and three minor loci. Notably, *QYr.CM62-2BL* was the preeminent QTL, explaining up to 50.59% of the phenotypic variance and exhibiting remarkable stability across 11 environments; it may represent a novel allele at the *QYR1* locus or a tightly linked QTL. Furthermore, we characterized *QYr.CM62-5BL*, a novel minor-effect QTL situated within the rearranged 5BL·7BL segment resulting from a 5B/7B reciprocal translocation present in both CM62 and the susceptible parent Avocet S. The cumulative effect of these additive QTL underpins the durable APR observed in CM62. To facilitate MAS, we developed two tightly linked KASP markers for *QYr.CM62-2BL*, which demonstrated high polymorphism in polymorphism analysis panels. Furthermore, in silico annotation of the *QYr.CM62-2BL* interval identified 35 high-confidence genes, including an NLR cluster and defense-related candidates, providing a solid foundation for future fine-mapping and functional validation of this locus. These findings not only advance our understanding of the resistance mechanisms in Chinese wheat germplasm but also provide robust molecular tools for the pyramiding of resistance alleles to enhance the durability and spectrum of stripe rust resistance in future wheat breeding programs.

## Figures and Tables

**Table 1:** The phenotypic variation in stripe rust disease severity across 13 environments and the corresponding mean values for the CM62/AvS RIL population.

Env ^a^	Parent			CM62/AvS RILs					
	CM62	AvS		Min ^b^	Max ^c^	Mean	Stdev ^d^	CV ^e^	*H^2^* ^f^
16XD	0.00	100.00		0	100	42.98	30.76	0.72	0.68
18XD	0.00	100.00		0	100	47.34	31.06	0.66	
19XD	0.00	100.00		0	100	33.27	35.71	1.07	
20XD	5.00	100.00		0	100	43.92	34.20	0.78	
21XD	0.00	100.00		0	100	53.11	31.29	0.59	
22XD	0.00	100.00		0	100	50.68	34.55	0.68	
21PX	0.00	100.00		0	100	41.06	35.78	0.87	
21MY	0.00	80.00		0	100	33.11	26.47	0.80	
21LS	5.00	80.00		0	100	33.30	23.97	0.72	
21JT	0.00	100.00		0	100	47.82	31.96	0.67	
22JT	0.00	100.00		0	100	44.43	31.91	0.72	
21XC	5.00	95.00		0	100	63.91	26.64	0.42	
22XC	0.00	80.00		0	95	35.10	23.96	0.68	
Mean	1.15	95.00		1.08	94.23	43.85	25.76	0.59	

^a^ Env, environment; The series 16XD, 18XD, 19XD, 20XD, 21XD, 22XD, 21PX, 21MY, 21LS, 21JT, 22JT, 21XC, and 22XC denote disease severity datasets evaluated from 13 field trial sites across the Xindu, Pixian, Mianyang, Linshui, Jitian, and Xichong districts of Sichuan Province, covering the period from 2016 to 2022. “Mean” indicates the mean disease severity across all 13 tested environments. ^b^ Min, minimum. ^c^ Max, maximum. ^d^ Stdev, standard deviation. ^e^ CV, coefficient of variation ^f^ *H^2^*, broad-sense heritability estimated across all 13 environments.

**Table 2:** Summary of stripe rust resistance QTL detected in the CM62/AvS RIL population across 13 environments and overall mean data.

QTL	Environment	Chromosome	Flanking Marker	Genetic Interval (cM)	Physical Position Interval (Mb) ^a^	LOD ^b^	PVE (%) ^c^	Add ^d^	QTL Comparison
*QYr.CM62-1BL*	16XD	1B	*AX-111240459*-*AX-111713183*	176.89–186.28	660.43–670.39	7.57	11.58	−10.56	*Yr29*
	18XD	1B	*AX-109350463*-*AX-111450994*	186.82–187.74	668.94–670.90	28.11	19.34	−14.12	
	21XC	1B	*AX-109350463*-*AX-111450994*	186.82–187.74	668.94–670.90	7.82	6.67	−6.67	
	22XC	1B	*AX-109350463*-*AX-111450994*	186.82–187.74	668.94–670.90	13.67	14.37	−8.83	
	21MY	1B	*AX-111450994*-*AX-108745708*	187.74–188.84	668.08–668.94	9.73	9.72	−8.05	
*QYr.CM62-2AS*	19XD	2A	*AX-110085687*-*AX-109887260*	0–0.56	0.29–17.54	4.73	2.98	−6.18	*Yr17/YrM1225*
	22XD	2A	*AX-110085687*-*AX-109887260*	0–0.55	0.29–17.54	18.78	8.13	−9.03	
	21PX	2A	*AX-110085687*-*AX-109887260*	0–0.56	0.29–17.54	13.49	6.61	−8.98	
	21MY	2A	*AX-110085687*-*AX-109887260*	0–0.56	0.29–17.54	9.52	9.38	−7.87	
	22JT	2A	*AX-110085687*-*AX-109887260*	0–0.56	0.29–17.54	15.90	15.17	−11.52	
	18XD	2A	*AX-110085687*-*AX-109973606*	0.55–2.45	0.29–24.88	28.53	20.01	−14.33	
	20XD	2A	*AX-110085687*-*AX-109973606*	0.55–2.45	0.29–24.88	16.56	7.28	−8.99	
	21LS	2A	*AX-110085687*-*AX-109973606*	0.55–2.45	0.29–24.88	8.62	11.04	−7.85	
	21JT	2A	*AX-110085687*-*AX-109973606*	0.55–2.45	0.29–24.88	20.75	19.63	−10.17	
	21XC	2A	*AX-110085687*-*AX-109973606*	0.55–2.45	0.29–24.88	15.14	13.98	−9.63	
	22XC	2A	*AX-110085687*-*AX-109973606*	0.55–2.45	0.29–24.88	15.66	16.73	−9.51	
	Mean	2A	*AX-110085687*-*AX-109973606*	0.55–2.45	0.29–24.88	28.35	13.89	−8.96	
*QYr.CM62-2BL*	20XD	2B	*AX-111497852*-*AX-110442665*	151.67–153.33	682.72–690.60	67.57	47.41	−22.86	*QYR1* region
	21PX	2B	*AX-111497852*-*AX-110442665*	151.67–153.33	682.72–690.60	63.49	49.61	−24.58	
	21XC	2B	*AX-111497852*-*AX-110442665*	151.67–153.33	682.72–690.60	22.40	21.41	−11.88	
	Mean	2B	*AX-111497852*-*AX-110442665*	151.67–153.33	682.72–690.60	57.23	36.90	−14.56	
	18XD	2B	*AX-111497852*-*AX-110442665*	151.67–153.33	682.72–690.60	14.65	8.97	−9.56	
	19XD	2B	*AX-111497852*-*AX-110442665*	151.67–153.33	682.72–690.60	53.35	50.59	−25.42	
	21XD	2B	*AX-111497852*-*AX-110442665*	151.67–153.33	682.72–690.60	64.08	49.13	−21.87	
	22XD	2B	*AX-111497852*-*AX-110442665*	151.67–153.33	682.72–690.60	60.84	38.31	−19.59	
	16XD	2B	*AX-111497852*-*AX-110442665*	151.67–153.33	682.72–690.60	9.39	12.66	−11.02	
	21MY	2B	*AX-111497852*-*AX-110442665*	151.67–153.33	682.72–690.60	22.66	25.34	−12.93	
	22JT	2B	*AX-109507945*-*AX-109421413*	153.88–154.06	691.99–696.35	17.63	17.09	−12.22	
	22XC	2B	*AX-109507945*-*AX-109421413*	153.88–154.06	691.99–696.35	13.20	13.59	−8.55	
*QYr.CM62-5AL.1*	20XD	5A	*AX-110058196*-*AX-111697333*	102.32–104.80	503.27–512.05	3.33	1.34	−3.86	*QYrsv.swust-5AL.1*
	21XD	5A	*AX-110058196*-*AX-111697333*	102.32–104.80	503.27–512.05	4.49	2.13	−4.57	
	21PX	5A	*AX-110058196*-*AX-111697333*	102.32–104.80	503.27–512.05	2.89	1.36	−4.09	
	22XD	5A	*AX-110427950*-*AX-111543503*	106.11–108.39	516.19–528.48	5.69	2.26	−4.78	
*QYr.CM62-5AL.2*	19XD	5A	*AX-109976129*-*AX-110947807*	138.87–140.85	565.55–566.18	4.40	2.84	−6.02	*Qyrsicau-5AL*
	18XD	5A	*AX-110947807*-*AX-111253877*	140.85–141.03	566.18–566.63	4.77	2.67	−5.21	
	22XD	5A	*AX-109933519*-*AX-110923044*	146.83–148.94	570.50–573.62	3.95	1.57	−3.96	
	Mean	5A	*AX-109933519*-*AX-110923044*	146.83–148.94	570.50–573.62	7.61	3.19	−4.28	
*QYr.CM62-5BL*	21XD	5BL·7BL	*AX-109480198*-*AX-110399021*	61.83–62.19	643.67–644.85	6.76	3.14	−5.55	Novel
	21JT	5BL·7BL	*AX-109480198*-*AX-110399021*	61.83–62.19	643.67–644.85	4.37	3.61	−4.36	
	20XD	5BL·7BL	*AX-109444987*-*AX-109963074*	65.35–66.47	622.55–626.57	7.31	3.01	−5.79	
	21XC	5BL·7BL	*AX-109444987*-*AX-109963074*	65.35–66.47	622.55–626.57	5.66	4.85	−5.69	
	Mean	5BL·7BL	*AX-109444987*-*AX-109963074*	65.35–66.47	622.55–626.57	5.73	2.38	−3.72	
	18XD	5BL·7BL	*AX-109444987*-*AX-109963074*	65.35–66.47	622.55–626.57	5.87	3.41	−5.92	

^a^ Physical position (Mb): the flanking marker sequences of QTL were blasted against IWGSC RefSeq V1.0 to get physical positions. ^b^ LOD, logarithm of odds. ^c^ PVE, phenotype variance explained. ^d^ Add, additive effect.

## Data Availability

The original contributions presented in this study are included in the article/Supplementary Material. Further inquiries can be directed to the corresponding author.

## Supplementary Materials

The following supporting information can be downloaded at: https://www.mdpi.com/article/10.3390/ijms27209196/s1.

## References

[1] Chen X.M. (2005). Epidemiology and control of stripe rust [*Puccinia Striiformis* f. Sp. *Tritici*] on wheat. Can. J. Plant Pathol..

[2] Chen X.M. (2020). Pathogens which threaten food security: *Puccinia Striiformis*, the wheat stripe rust pathogen. Food Secur..

[3] Wang M.N., Chen X.M., Chen X.M., Kang Z.S. (2017). Stripe rust resistance. Stripe Rust.

[4] Chen W.Q., Kang Z.S., Ma Z.H., Xu S.C., Jin S.L., Jiang Y.Y. (2013). Integrated management of wheat stripe rust caused by *Puccinia striiformis* f. sp. *tritici* in China. Sci. Agric. Sin..

[5] Wan A.M., Chen X.M., Yuen J. (2016). Races of *Puccinia Striiformis* f. Sp. *Tritici* in the united states in 2011 and 2012 and comparison with races in 2010. Plant Dis..

[6] Chen W.Q., Liu T.G. (2023). Occurrence of wheat stripe rust in autumn-sown seedlings and inter-regional inoculum dispersal of *Puccinia striiformis* f. Sp. *tritici* in China. Sci. Agric. Sin..

[7] Chen X.M. (2013). Review Article: High-temperature adult-plant resistance, key for sustainable control of stripe rust. Am. J. Plant Sci..

[8] McIntosh R.A., Dubcovsky J., Rogers W.J., Morris C., Appels R., Xia X.C. Catalogue of Gene Symbols for Wheat. GrainGenes, 2023. https://wheat.pw.usda.gov/GG3/wgc.

[9] Park R.F., Chhetri M., Sandhu K.S., Ziems L., Singh D. (2026). Discovery and mapping of a new gene in wheat for race-specific adult plant resistance to *Puccinia striiformis* f. sp. *tritici*. Theor. Appl. Genet..

[10] Yao F.J., Yang M.Y., Li J., Yang N., Gan X.Q., Yang W.Y., Yang E.N. (2026). mapping and validation of a novel major QTL for adult plant-stage resistance to stripe rust in the wheat landrace ‘Xuxumai’. Phytopathology.

[11] Wellings C.R. (2011). Global status of stripe rust: A review of historical and current threats. Euphytica.

[12] Wang F., Zhang M.H., Hu Y.L., Gan M.J., Jiang B., Hao M., Ning S.Z., Liu D.C., Huang L. (2023). Pyramiding of adult-plant resistance genes enhances all-stage resistance to wheat stripe rust. Plant Dis..

[13] Davis H.R., Chia T., Rhodes H., Leaman C., Lewis C.M., Savva L., Korolev A., Boyd L., Saunders D.G.O., Nellist C.F. (2025). First large-scale breakdown of *Yr15* resistance to wheat yellow rust (*Puccinia striiformis* f. sp. *tritici*) recorded globally. New Dis. Rep..

[14] Hansen J.G. (2025). Yr15-Virulence in Yellow Rust Confirmed in Continental Europe.

[15] Liu Z.Y., Zhang H.Z., Bai B., Li J., Huang L., Xu Z.B., Chen Y.X., Liu X., Cao T.J., Li M.M. (2024). Current status and strategies for utilization of stripe rust resistance genes in wheat breeding program of China. Sci. Agric. Sin..

[16] Singh R.P., Huerta-Espino J., Bhavani S., Herrera-Foessel S.A., Singh D., Singh P.K., Velu G., Mason R.E., Jin Y., Njau P. (2011). Race non-specific resistance to rust diseases in CIMMYT spring wheats. Euphytica.

[17] He Z.H., Lan C.X., Chen X.M., Zou Y.C., Zhuang Q.S., Xia X.C. (2011). Progress and perspective in research of adult-plant resistance to stripe rust and powdery mildew in wheat. Sci. Agric. Sin..

[18] Moore J.W., Herrera-Foessel S., Lan C., Schnippenkoetter W., Ayliffe M., Huerta-Espino J., Lillemo M., Viccars L., Milne R., Periyannan S. (2015). A recently evolved hexose transporter variant confers resistance to multiple pathogens in wheat. Nat. Genet..

[19] Wang Z., Ren J.D., Du Z.Y., Che M.Z., Zhang Y.B., Quan W., Jiang X., Ma Y., Zhao Y., Zhang Z.J. (2019). Identification of a major QTL on chromosome arm 2AL for reducing yellow rust severity from a Chinese wheat landrace with evidence for durable resistance. Theor. Appl. Genet..

[20] Lin M., Dieseth J.A., Alsheikh M., Yang E., Holzapfel J., Schürmann F., Morales L., Michel S., Buerstmayr H., Bhavani S. (2023). A major yellow rust resistance QTL on chromosome 6A shows increased frequency in recent Norwegian spring wheat cultivars and breeding lines. Theor. Appl. Genet..

[21] Ye X.L., Li J., Cheng Y.K., Yao F.J., Long L., Yu C., Wang Y.Q., Wu Y., Li J., Wang J.R. (2019). Genome-wide association study of resistance to stripe rust (*Puccinia striiformis* f. sp. *tritici*) in Sichuan wheat. BMC Plant Biol..

[22] Li J., Jiang Y.F., Yao F.J., Long L., Wang Y.Q., Wu Y., Li H., Wang J.R., Jiang Q.T., Kang H.Y. (2020). Genome-wide association study reveals the genetic architecture of stripe rust resistance at the adult plant stage in Chinese endemic wheat. Front. Plant Sci..

[23] Hao Y.F., Chen Z.B., Wang Y.Y., Bland D., Buck J., Brown-Guedira G., Johnson J. (2011). Characterization of a major QTL for adult plant resistance to stripe rust in US soft red winter wheat. Theor. Appl. Genet..

[24] Dhillon G.S., Kaur S., Das N., Singh R., Poland J., Kaur J., Chhuneja P. (2020). QTL mapping for stripe rust and powdery mildew resistance in *Triticum durum–Aegilops speltoides* backcross introgression lines. Plant Genet. Resour..

[25] Tang Z.X., Zhang R.Y., Gong S.J., Li G.R., Yang Z.J., Dong W.T., Fu S.L. (2026). Development and characterization of a new wheat-rye T6BS.6BL-6RL^ku^ small segment translocation with powdery mildew resistance and potential breeding value. Crop J..

[26] Yang M.Y., Yang Z.J., Yang W.Y., Yang E.N. (2018). Development and identification of new wheat varieties (lines) with multiple translocation chromosomes via cytogenetic method. J. Triticeae Crops.

[27] Lillemo M., Asalf B., Singh R.P., Huerta-Espino J., Chen X.M., He Z.H., Bjørnstad Å. (2008). The adult plant rust resistance loci *Lr34/Yr18* and *Lr46/Yr29* are important determinants of partial resistance to powdery mildew in bread wheat line Saar. Theor. Appl. Genet..

[28] Singh A., Pandey M.P., Singh A.K., Knox R.E., Ammar K., Clarke J.M., Clarke F.R., Singh R.P., Pozniak C.J., DePauw R.M. (2013). Identification and mapping of leaf, stem and stripe rust resistance quantitative trait loci and their interactions in durum wheat. Mol. Breed..

[29] Cobo N., Wanjugi H., Lagudah E., Dubcovsky J. (2019). A high-resolution map of wheat *QYr.ucw-1BL*, an adult plant stripe rust resistance locus in the same chromosomal region as *Yr29*. Plant Genome.

[30] Bariana H.D., Mcintosh R.A. (1993). Cytogenetic studies in wheat. XV. Location of rust resistance genes in VPMl and their genetic linkage with other disease resistance genes in chromosome 2A. Genome.

[31] Liu L., Wang M.N., Zhang Z.W., See D.R., Chen X.M. (2020). Identification of stripe rust resistance loci in U.S. spring wheat cultivars and breeding lines using genome-wide association mapping and *Yr* gene markers. Plant Dis..

[32] Mu J.M., Liu L., Liu Y., Wang M.N., See D.R., Han D.J., Chen X.M. (2020). Genome-wide association study and gene specific markers identified 51 genes or qtl for resistance to stripe rust in U.S. winter wheat cultivars and breeding lines. Front. Plant Sci..

[33] Li Y.X., Liu L., Wang M.N., Ruff T., See D.R., Hu X.P., Chen X.M. (2023). Characterization and molecular mapping of a gene conferring high-temperature adult-plant resistance to stripe rust originally from *Aegilops Ventricosa*. Plant Dis..

[34] Marchal C., Zhang J., Zhang P., Fenwick P., Steuernagel B., Adamski N.M., Boyd L., McIntosh R., Wulff B.B.H., Berry S. (2018). BED-domain-containing immune receptors confer diverse resistance spectra to yellow rust. Nat. Plants.

[35] Boukhatem N., Baret P.V., Mingeot D., Jacquemin J.M. (2002). Quantitative trait loci for resistance against yellow rust in two wheat-derived recombinant inbred line populations. Theor. Appl. Genet..

[36] Zhou X., Zhong X., Roter J., Li X., Yao Q., Yan J., Yang S., Guo Q., Distelfeld A., Sela H. (2021). Genome-wide mapping of loci for adult-plant resistance to stripe rust in durum wheat Svevo using the 90k SNP Array. Plant Dis..

[37] Long L., Yao F.J., Guan F.N., Cheng Y.K., Duan L.Y., Zhao X.Y., Li H., Pu Z.E., Li W., Jiang Q.T. (2021). A stable quantitative trait locus on chromosome 5BL combined with *Yr18* conferring high-level adult plant resistance to stripe rust in Chinese wheat landrace Anyuehong. Phytopathology.

[38] Lu Y., Lan C., Liang S., Zhou X., Liu D., Zhou G., Lu Q., Jing J., Wang M., Xia X. (2009). QTL Mapping for adult-plant resistance to stripe rust in italian common wheat cultivars Libellula and Strampelli. Theor. Appl. Genet..

[39] Bansal U.K., Kazi A.G., Singh B., Hare R.A., Bariana H.S. (2014). Mapping of durable stripe rust resistance in a durum wheat cultivar Wollaroi. Mol. Breed..

[40] Riley R., Coucoli H., Chapman V. (1967). Chromosomal interchanges and the phylogeny of wheat. Heredity.

[41] Law C.N., Worland A.J. (1997). The control of adult-plant resistance to yellow rust by the translocated chromosome 5BS-7BS of bread wheat. Plant Breed..

[42] Mallard S., Gaudet D., Aldeia A., Abelard C., Besnard A.L., Sourdille P., Dedryver F. (2005). Genetic analysis of durable resistance to yellow rust in bread wheat. Theor. Appl. Genet..

[43] Badaeva E.D., Dedkova O.S., Gay G., Pukhalskyi V.A., Zelenin A.V., Bernard S., Bernard M. (2007). Chromosomal rearrangements in wheat: Their types and distribution. Genome.

[44] Yao F.J., Guan F.N., Duan L.Y., Long L., Tang H., Jiang Y.F., Pu Z.E., Deng M., Wei Y.M., Zheng Y.L. (2021). Genome-wide association analysis of stable stripe rust resistance loci in a Chinese wheat landrace panel using the 660K SNP array. Front. Plant Sci..

[45] Liu L., Wang M.N., Feng J.Y., See D.R., Chen X.M. (2019). Whole-genome mapping of stripe rust resistance quantitative trait loci and race specificity related to resistance reduction in winter wheat cultivar Eltan. Phytopathology.

[46] Yao F.J., Wang M., See D.R., Yang E.N., Chen G.Y., Chen X.M. (2024). Identification of 39 stripe rust resistance loci in a panel of 465 winter wheat entries presumed to have high-temperature adult-plant resistance through genome-wide association mapping and marker-assisted detection. Front. Plant Sci..

[47] Yao F.J., Long L., Wang Y.Q., Duan L.Y., Zhao X.Y., Jiang Y.F., Li H., Pu Z.E., Li W., Jiang Q.T. (2020). Population structure and genetic basis of the stripe rust resistance of 140 Chinese wheat landraces revealed by a genome-wide association study. Plant Sci..

[48] Yao F.J., Zhang X.M., Ye X.L., Li J., Long L., Yu C., Li J., Wang Y.Q., Wu Y., Wang J.R. (2019). Characterization of molecular diversity and genome-wide association study of stripe rust resistance at the adult plant stage in northern Chinese wheat landraces. BMC Genet..

[49] Liu S., Liu D., Zhang C., Zhang W., Wang X., Mi Z., Gao X., Ren Y., Lan C., Liu X. (2023). Slow stripe rusting in Chinese wheat Jimai 44 conferred by *Yr29* in combination with a major QTL on chromosome arm 6AL. Theor. Appl. Genet..

[50] Yang M.Y., Li G.R., Wan H.S., Li L.P., Li J., Yang W.Y., Pu Z.J., Yang Z.J., Yang E.N. (2019). Identification of QTLs for stripe rust resistance in a recombinant inbred line population. Int. J. Mol. Sci..

[51] Peterson R.F., Campbell A.B., Hannah A.E. (1948). A diagrammatic scale for estimating rust intensity on leaves and stems of cereals. Can. J. Res..

[52] Wickham H. (2009). Ggplot2: Elegant Graphics for Data Analysis.

[53] Wei T.Y., Simko V., Levy M., Xie Y.H., Jin Y., Zemla J., Freidank M., Cai J., Protivinsky T. (2024). R Package Corrplot: Visualization of a Correlation Matrix (v0.95). https://CRAN.R-project.org/package=corrplot.

[54] Gu Z.G., Gu L., Eils R., Schlesner M., Brors B. (2014). *Circlize* implements and enhances circular visualization in R. Bioinformatics.

[55] Clarke J. (2009). Cetyltrimethyl Ammonium Bromide (CTAB) DNA miniprep for plant DNA isolation. Cold Spring Harb. Protoc..

[56] Fu S.L., Chen L., Wang Y.Y., Li M., Yang Z.J., Qiu L., Yan B.J., Ren Z.L., Tang Z.X. (2015). Oligonucleotide probes for ND-FISH analysis to identify rye and wheat chromosomes. Sci. Rep..

[57] Yang M.Y., Yang Z.J., Yang W.Y., Yang E.N. (2022). Genetic diversity assessment of the international maize and wheat improvement center and Chinese wheat core germplasms by non-denaturing fluorescence in situ hybridization. Plants.

[58] Ma S.W., Wang M., Wu J.H., Guo W.L., Chen Y.M., Li G.W., Wang Y.P., Shi W.M., Xia G.M., Fu D.L. (2021). WheatOmics: A platform combining multiple omics data to accelerate functional genomics studies in wheat. Mol. Plant..

